# Food-Related Online Media Viewing (*Mukbang* and *Cookbang*) and Sugar-Sweetened Beverage and Artificially Sweetened Beverage Consumption Among Korean Adolescents: Using the 2025 Korea Youth Risk Behavior Survey

**DOI:** 10.3390/nu18203402

**Published:** 2026-10-16

**Authors:** Soo Jin Lee, Seol Hwa Park, Cynthia Y. Yoon

**Affiliations:** 1 Department of Food Science and Nutrition, Pusan National University, Busan 46241, Republic of Korea; 2 BK21 FOUR Program, Precision Nutrition Program for Future Global Leaders, Pusan National University, Busan 46241, Republic of Korea; 3 Kimchi Research Institute, Pusan National University, Busan 46241, Republic of Korea

**Keywords:** adolescents, media, sugar-sweetened beverage, artificially sweetened beverage, Korea youth risk behavior survey

## Abstract

**Background/Objectives:** Adolescents are increasingly exposed to food-related online media, with *mukbang* and *cookbang* representing prominent forms of such media. *Mukbang* refers to an online genre in which hosts consume food while interacting with viewers, whereas *cookbang* refers to an online genre in which hosts prepare and often consume food. In this study, these genres are termed as food-related online media. Adolescents who frequently view food-related online media have been reported to consume more energy-dense foods, with differences by sex and age. However, little is known about their association with beverage consumption. This study examined the associations between food-related online media viewing and consumption of sugar-sweetened beverages (SSBs) and artificially sweetened beverages (ASBs) among adolescents and whether these associations differed by sex and school level. **Methods:** Data were drawn from the 2025 Korea Youth Risk Behavior Survey (*n* = 27,104; M_age_ = 14.98 ± 1.78 years; 40.9% girls; 50.7% middle school students). Food-related online media viewing and SSB and ASB consumption frequencies were self-reported. Multinomial logistic regression models examined associations of food-related online media viewing with SSB and ASB consumption, stratified by sex and school level. **Results:** Frequent viewing of such media (≥5 times/week) was associated with greater odds of SSB consumption ≥ 7 times/week than infrequent food-related online media viewing (<once/month). The association was stronger among boys (OR = 1.79, 95% CI = 1.39–2.31) than girls (OR = 1.37, 95% CI = 1.14–1.65) and among middle school students (OR = 2.07, 95% CI = 1.66–2.57) than high school students (OR = 1.28, 95% CI = 1.05–1.56). Associations with ASB consumption were generally stronger among boys than girls and varied across consumption frequencies and school levels. **Conclusions:** Frequent viewing of food-related online media was associated with adolescent’s SSB and ASB consumption, although it differed by sex and school level. These findings underscore the importance of considering differences across adolescent subgroups when developing strategies to reduce SSB and ASB consumption.

## 1. Introduction

Adolescence is a critical developmental period during which youth engage extensively with digital media [1,2]. Although digital media encompass a wide range of content, food-related online media has become increasingly popular [3,4,5,6]. Among these, *mukbang* and *cookbang* have emerged as prominent forms of food-related media content in Korea. *Mukbang* typically presents hosts consuming food while interacting with viewers, whereas *cookbang* generally features hosts preparing food and, in many cases, consuming it. Although these *mukbang* and *cookbang* differ in their primary content and viewer experiences, both are characterized by prominent food-related visual and social cues and provide viewers with repeated exposure to food preparation, presentation, and consumption. Accordingly, the Korea Youth Risk Behavior Survey (KYRBS) assesses the viewing of *mukbang* and *cookbang* jointly as a measure of “food-related online media.”

Viewing food-related online media is particularly common among Korean adolescents [3,4,5,6]. Nationally representative data report that more than two-thirds of Korean adolescents have viewed *mukbang*/*cookbang*, with a substantial proportion of adolescents reporting frequent viewing (≥7 times/week) [6,7]. The high prevalence of this exposure has raised public health concerns because such content often features large portions of highly palatable, energy-dense foods [3,8]. Food-related online media may also provide salient food cues and portray eating and food preparation as social or entertaining activities. Adolescents may be particularly responsive to such content because of developmental changes in susceptibility to social modeling and persuasive media messages [9,10]. Consistent with this, observational studies have reported associations between food-related online media viewing and several food-related behaviors, including food choices [11,12,13], late-night eating [14], use of food delivery services [14], appetite [15], and impulsive food purchasing [16]. Despite this growing body of studies reporting adverse consequences of food-related online media, most research thus far has focused primarily on food choices and behaviors [11,12,13], with less attention given to beverage consumption [11,12,17].

Understanding whether food-related online media viewing is associated with beverage consumption is important because beverages comprise a major component of adolescents’ diets and contribute substantially to overall diet quality, energy intake, and cardiometabolic health [18,19]. Sugar-sweetened beverage (SSB) consumption among adolescents remains high worldwide, with nearly two-thirds reporting SSB consumption [20,21]. High SSB consumption is a major public health concern, because it is a source of added sugars and has been associated with obesity, insulin resistance, type 2 diabetes, cardiovascular disease, and dental caries [22,23,24]. Because food-related online media frequently depicts highly palatable foods together with SSBs [12,25], viewing such media may reinforce favorable perceptions of these beverages and increase their appeal. Nevertheless, whether such exposure is associated with adolescents’ SSB consumption remains unclear. To date, few studies have examined the association between food-related online media viewing and SSB consumption among adolescents [11,12,17].

Artificially sweetened beverages (ASBs), often marketed as “zero-sugar” or “zero-calorie” alternatives to SSBs, have become increasingly popular among adolescents [26,27,28,29,30]. Although ASBs are commonly perceived as healthier beverage choices, evidence regarding their long-term health effects remains inconclusive [26,31,32]. Some studies have reported short-term benefits, such as weight loss or weight management [29], whereas others have found no benefit or have reported adverse long-term health outcomes [30,33]. Because food-related online media may portray both highly palatable foods, sugar-containing beverages, and artificially sweetened beverages, repeatedly watching such media may influence beverage choices through both reward-driven and reflective processes. From a dual-process perspective [34] on eating behavior, exposure to food cues may be relevant to both hedonic responses to palatable foods and reflective considerations of sugar or energy content. These processes could plausibly be related to consumption of either SSBs or ASBs, although observational studies cannot establish whether food-related online media exposure leads to changes in beverage choice. Despite emerging evidence suggesting an association between food-related online media viewing and SSB consumption [11,12,17], to our knowledge, no previous study has examined the association between food-related online media viewing and ASB consumption among adolescents.

Because general media use, particularly exposure to online media, beverage consumption, and body image concerns differ by sex and developmental stage [7,35,36,37,38,39] and may persist into adulthood [40,41,42], associations between food-related online media viewing and beverage consumption may also differ across adolescent subgroups. Girls generally report greater body image concerns and dieting behaviors [37], whereas boys often consume greater quantities of SSBs. Similarly, compared with middle school students, high school students are approaching a developmental period characterized by greater body image concerns, dieting behaviors, and disordered eating behaviors [41,42,43], which may be associated with an increased interest in beverages perceived as lower in sugar or calories [41]. Examining these subgroup differences may help identify adolescents who are particularly susceptible to the influence of food-related online media.

Therefore, this study examined the associations between the KYRBS-defined, food-related online media exposure (*mukbang/cookbang* viewing) and SSB and ASB consumption among Korean adolescents using nationally representative data from the Korea Youth Risk Behavior Survey. We further examined whether these associations differ by sex and school level. We hypothesized that more frequent food-related online media viewing would be associated with higher odds of both SSB and ASB consumption and that the association between food-related online media viewing and SSB consumption would be stronger among boys and middle-school students, while the association with ASB consumption would be stronger among girls and high-school students.

## 2. Materials and Methods

### 2.1. Study Sample and Data

**Figure 1:**
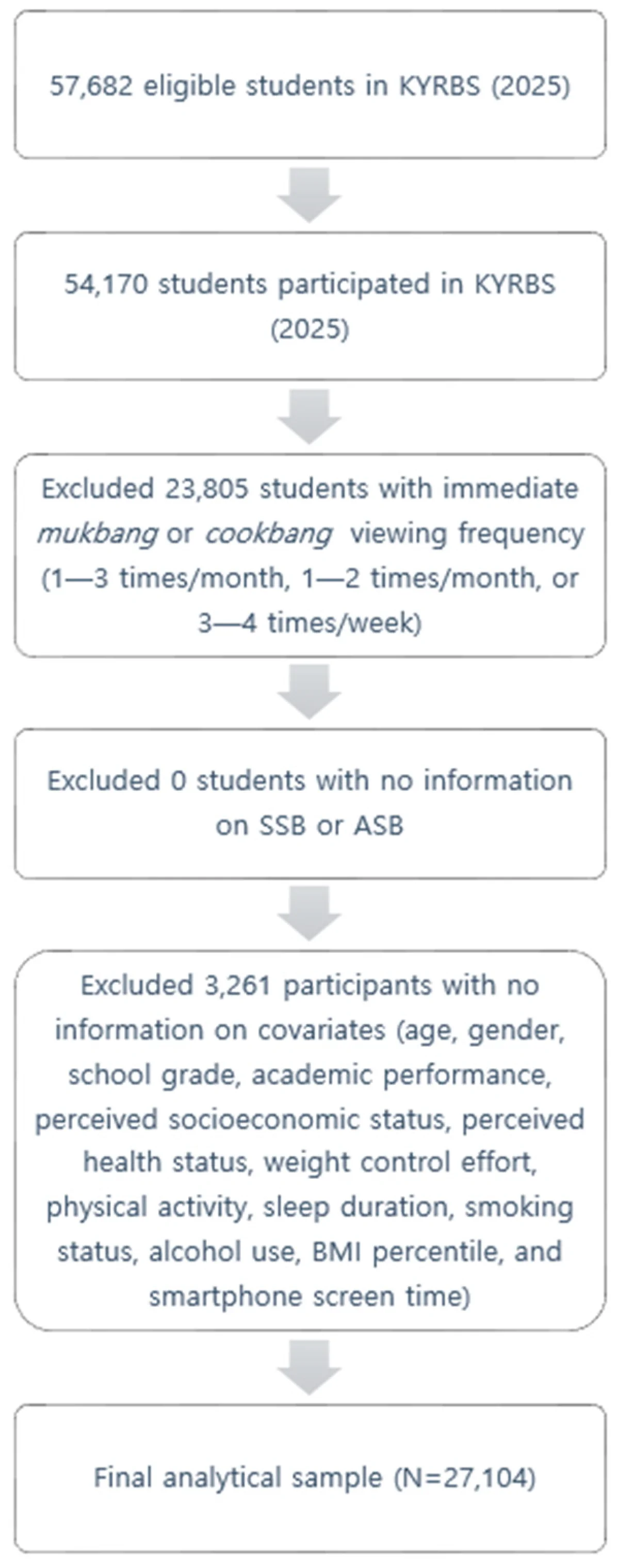
Flow chart.

Data were obtained from the 2025 Korea Youth Risk Behavior Survey (KYRBS), the 21st wave of a nationally representative repeated cross-sectional survey of Korean adolescents aged 12–18 years [43]. Participants completed an anonymous, self-administered online questionnaire [43]. Detailed survey methods have been described elsewhere [43]. Of the 57,682 students selected to participate in the 2025 KYRBS, 54,170 responded (response rate: 93.9%). After excluding participants with intermediate viewing of food-related online media (i.e., those who viewed *mukbang* and/or *cookbang* between 1 and 4 times per week) (*n* = 23,805) and missing covariate data (*n* = 3261), the final analytical sample included 27,104 adolescents (Figure 1). The KYRBS protocol was approved by the Korea Disease Control and Prevention Agency and conducted in accordance with the Declaration of Helsinki. The present study was reviewed by the Institutional Review Board (IRB) of Pusan National University and was determined to be exempt (IRB No. 2026-07-028).

### 2.2. Measures

#### 2.2.1. Independent Variable

Food-related online media viewing was assessed using the KYRBS item that asks participants about their frequency of viewing *mukbang* or *cookbang*. Because the KYRBS assesses *mukbang* and *cookbang* jointly, the exposure was operationalized as combined *mukbang*/*cookbang* viewing frequency and referred to as “food-related online media viewing” in the present study. Food-related online media viewing was assessed using a single item asking participants how often they had watched *mukbang* or *cookbang* during the previous 12 months. Response options were “not at all,” “less than once a month,” “1–3 times per month,” “1–2 times per week,” “3–4 times per week,” “5–6 times per week,” and “daily.” Participants were categorized as frequent viewers (5–6 times per week or daily) or infrequent viewers (less than once a month or not at all). Participants reporting intermediate viewing frequencies (1–3 times per month, 1–2 times per week, or 3–4 times per week) were excluded to maximize the contrast between exposure groups (Table 1).

#### 2.2.2. Dependent Variable

SSB and ASB consumption were assessed using separate questions from the 2025 KYRBS. The KYRBS is a nationally representative surveillance survey conducted annually by the Korea Disease Control and Prevention Agency to monitor health-related behaviors among Korean adolescents.

Sugar-Sweetened Beverage (SSB) Consumption

SSB consumption was assessed by asking “In the past 7 days, how often did you drink sweetened beverages?” The survey provided examples of sweetened beverages, including carbonated beverages (excluding zero beverages), energy drinks (e.g., Hot6, Red Bull, and Bacchus), sports/isotonic drinks (e.g., Gatorade and Pocari Sweat), fruit beverages (e.g., SunKist Drink and Capri-Sun), coffee beverages (e.g., coffee mix and Let’s Be), and sweetened milk (e.g., chocolate and banana milk).

Artificially Sweetened Beverage (ASB) Consumption

ASB consumption was assessed using the survey-defined ‘zero beverage’ category. The 2025 Korea Youth Risk Behavior Survey defined zero beverages as beverages that use artificial sweeteners instead of sugar to provide sweetness but contain almost no calories. Examples included Coke Zero, Sprite Zero, Powerade Zero, and Toreta Zero.

For both SSB and ASB consumption, participants reported their frequency of consumption during the previous 7 days. Similar single-item beverage-frequency questions have been routinely used in the KYRBS to assess beverage consumption among adolescents [25,32]. Because the item was designed for population-level behavioral surveillance, it assessed frequency of consumption rather than the amount or volume consumed. The specific response categories and coding used in the present analysis are provided in Table 1.

### 2.3. Moderators

Sex (male/female) and school level (middle school [grades 1–3] vs. high school [grades 1–3]) were examined as moderators (Table 1).

### 2.4. Covariates

Covariates included age, academic performance, perceived socioeconomic status, perceived health, weight-control effort, physical activity, sleep duration, smoking status, alcohol use, BMI percentile, and smartphone screen time [44]. Sex was included as a covariate in models examining school-level differences in the associations of food-related online media viewing with SSB and ASB consumption. Categorical covariates were coded according to the categories presented in Table 1. BMI percentile was classified according to the 2017 Korean National Growth Charts for Children and Adolescents. Average sleep duration was calculated as the 7-day average using weekday and weekend sleep duration: [(weekday sleep duration × 5) + (weekend sleep duration × 2)]/7, following a previously reported approach [45]. Average smartphone screen time was calculated as the 7-day average using self-reported smartphone use on weekdays and weekends: [(weekday average smartphone usage time × 5) + (weekend average smartphone usage time × 2)]/7, consistent with a previous study using the Korea Youth Risk Behavior Survey [46]. Covariates were selected based on previous literature and their potential association between food-related online media viewing and beverage consumption.

### 2.5. Statistical Analysis

All analyses accounted for the complex sampling design of the KYRBS, which employed stratified multistage cluster sampling and sampling weights. Participant characteristics were compared across food-related online media viewing groups using the Rao–Scott chi-square test for categorical variables and a complex-sample general linear model for continuous variables. Sex- and school level- stratified associations between food-related online media viewing and beverage consumption were examined using multinomial logistic regression to generate odds ratios (ORs) and 95% confidence intervals (95% CIs). This multinomial logistic regression was used as the primary analytical approach because it does not impose the proportional-odds assumption and allows associations to vary across beverage-consumption frequency categories. For both SSB and ASB consumption, the “never” consumption category was specified as the reference outcome category. Model 1 was unadjusted, while Model 2 was adjusted for age, sex (for school-level stratified analyses), academic performance, perceived socioeconomic status, perceived health status, weight-control effort, physical activity, sleep duration, smoking status, alcohol use, BMI percentile, and smartphone screen time.

For the primary analyses, participants were categorized as infrequent or frequent viewers, with intermediate viewers excluded. The two viewing groups were analyzed as subpopulations within the complex survey framework, while retaining the original sampling weights, strata, and primary sampling units for variance estimation. This approach preserved the original complex sampling design when estimating survey-based standard errors. Effect modification by sex and school level was assessed by including multiplicative interaction terms in the regression models. Based on a priori hypotheses regarding differences by sex and school level, stratified analyses were conducted to further examine associations within these prespecified subgroups.

Sensitivity analyses were conducted to assess the robustness of the findings to the categorization of food-related online media viewing and the choice of regression model. First, analyses including intermediate viewers as a separate category (infrequent, intermediate, and frequent) were conducted (Supplementary Tables S1 and S2). Second, given that the proportional-odds assumption was violated only for SSB consumption among high school students, ordinal logistic regression using a proportional-odds model was conducted in place of the multinomial logistic regression used in the primary analysis (Supplementary Table S3). All analyses were conducted using SPSS version 30.0 and SAS version 9.4.

## 3. Results

### 3.1. General Characteristics of Study Participants

The analytic sample comprised 27,104 adolescents. Among the 27,104 adolescents, 4354 were classified as adolescents who frequently viewed food-related online media (≥5 times/week) whereas 22,750 (83.9%) were classified as infrequent viewers (≤once/month). Overall, 59.1% were boys, the mean age was 14.98 years, and 50.7% were middle school students. Details of the general characteristics of the study participants by food-related online media viewing frequency are shown in Table 2.

### 3.2. Effect Modification by Sex and School Level

In interaction analyses, significant effect modification was observed by sex for SSB consumption (*p*-interaction = 0.014) and ASB consumption (*p*-interaction = 0.040) and by school level for SSB consumption (*p*-interaction < 0.001). The interaction between food-related online media viewing and school level for ASB consumption was not statistically significant (*p*-interaction = 0.165). However, because school level was a prespecified subgroup variable and our a priori hypothesis was that associations with ASB consumption would be stronger among high school students, school-level-stratified estimates are presented to describe associations within each school-level subgroup.

### 3.3. Association Between Food-Related Online Media Viewing and Sugar-Sweetened Beverage Consumption Frequency, Stratified by Sex

#### 3.3.1. Boys

Boys who frequently viewed food-related online media had ORs of 1.28 (95% CI = 0.99–1.64) and 1.79 (95% CI = 1.40–2.28) for consuming SSB 5–6 and ≥7 times per week, respectively, compared with infrequent viewers. After adjustment, the corresponding ORs were 1.38 (95% CI = 1.06–1.79) and 1.79 (95% CI = 1.39–2.31), respectively (Table 3, Model 2). No statistically significant associations were observed between for SSB consumption 1–2 or 3–4 times per week regardless of the level of adjustment (Table 3, Models 1,2).

#### 3.3.2. Girls

Among girls, frequent food-related online media viewing was associated with higher odds of consuming SSB 3–4 times per week (OR = 1.21, 95% CI = 1.03–1.41), 5–6 times per week (OR = 1.60, 95% CI = 1.32–1.92) and ≥7 times per week (OR = 1.57, 95% CI = 1.31–1.88) in Model 1. After adjustment, the associations for 5–6 times and ≥7 times per week remained statistically significant, with ORs of 1.44 (95% CI = 1.19–1.74) and 1.37 (95% CI = 1.14–1.65), respectively, whereas the association for 3–4 times was attenuated and no longer statistically significant (OR = 1.11, 95% CI = 0.95–1.31) (Table 3, Model 2).

### 3.4. Association Between Food-Related Online Media Viewing and Artificially Sweetened Beverage Consumption, Stratified by Sex

#### 3.4.1. Boys

Boys who frequently viewed food-related online media had higher odds of ASB consumption at all frequencies in Model 1, with ORs ranging from 1.46 (95% CI = 1.26–1.69) for 1–2 times per week to 3.86 (95% CI = 3.08–4.82) for ≥7 times per week. (Table 3, Model 1). After adjustments, the associations remained statistically significant across all consumption frequencies, with ORs of 1.36 (95% CI = 1.16–1.59), 2.02 (95% CI = 1.67–2.46), 2.37 (95% CI = 1.84–3.06), and 3.98 (95% CI = 3.23–4.91), for 1–2 times, 3–4 times, 5–6 times, and ≥7 times per week, respectively (Table 3, Model 2).

#### 3.4.2. Girls

Among girls, frequent food-related online media viewing was associated with higher odds of ASB consumption across all frequencies in Model 1, with ORs ranging from 1.34 (95% CI = 1.22–1.47) for 1–2 times per week to 2.44 (95% CI = 1.88–3.16) for ≥7 times per week (Table 3, Model 1). After adjustments, the associations remained statistically significant, although the estimates were attenuated, with adjusted ORs ranging from 1.24 to 2.20 (Table 3, Model 2).

### 3.5. Association Between Food-Related Online Media Viewing and Sugar-Sweetened Beverage Consumption Frequency, Stratified by School Level

#### 3.5.1. Middle School

Among middle school students, frequent food-related online media viewing was associated with higher odds of consuming SSB 5–6 times per week (OR = 1.50, 95% CI = 1.20–1.88), and ≥7 times per week (OR = 1.85, 95% CI = 1.52–2.26) (Table 4, Model 1). After adjustments, these associations accentuated to 1.80 (95% CI = 1.41–2.30) and 2.07 (95% CI = 1.66–2.57), respectively (Table 4, Model 2).

#### 3.5.2. High School

Among high school students, frequent food-related online media viewing was inversely associated with SSB consumption 1–2 times per week (OR = 0.82, 95% CI = 0.70–0.96, Table 4, model 1), while no positive associations were observed for the higher consumption frequencies. After adjustments, the associations for 1–2 times per week was attenuated and no longer statistically significant (OR = 0.86, 95% CI = 0.72–1.02), whereas frequent viewing was associated with higher odds of consuming SSB ≥ 7 times per week (OR = 1.28, 95% CI = 1.05–1.56) (Table 4, model 2).

### 3.6. Association Between Food-Related Online Media Viewing and Artificially Sweetened Beverage Consumption, Stratified by School Level

#### 3.6.1. Middle School

Among middle school students, frequent food-related online media viewing was associated with higher odds of ASB consumption across all frequencies in Model 1, with ORs ranging from 1.22 (95% CI = 1.09–1.36) for 1–2 times per week to 2.53 (95% CI = 2.00–3.20) for ≥7 times per week (Table 4, Model 1). After adjustments, the associations remained statistically significant, with adjusted ORs of 1.26 (95% CI = 1.12–1.42), 1.43 (95% CI = 1.19–1.70), 1.66 (95% CI = 1.23–2.21), and 3.45 (95% CI = 2.77–4.31), for 1–2 times, 3–4 times, 5–6 times, and ≥7 times per week, respectively (Table 4, Model 2).

#### 3.6.2. High School

Among high school students, frequent food-related online media viewing was associated with higher odds of ASB consumption across all frequencies in Model 1, with ORs ranging from 1.29 (95% CI = 1.15–1.45) for 1–2 times per week to 1.90 (95% CI = 1.53–2.36) for ≥7 times per week (Table 4, Model 1). After adjustment, the associations remained significant, with ORs of 1.28 (95% CI = 1.13–1.44), 1.92 (95% CI = 1.64–2.26), 1.93 (95% CI = 1.52–2.46), and 2.28 (95% CI = 1.81–2.88), for 1–2 times, 3–4 times, 5–6 times, and ≥7 times per week, respectively (Table 4, Model 2).

### 3.7. Sensitivity Analyses

Sensitivity analyses were conducted to assess the robustness of the findings to the categorization of food-related online media viewing and the choice of regression model. First, analyses including intermediate viewers as a separate category (infrequent, intermediate, and frequent) were conducted (Supplementary Tables S1 and S2). When intermediate viewers were included as a separate category, intermediate viewers showed significantly higher odds of reporting more frequent SSB and ASB consumption than infrequent viewers across several consumption categories and demographic strata (Supplementary Tables S1 and S2). Associations for frequent viewers were generally consistent in direction with those observed in the primary analysis, although some additional statistically significant associations were observed. Second, ordinal logistic regression using a proportional-odds model was conducted in place of the multinomial logistic regression used in the primary analysis (Supplementary Table S3). Frequent viewers had higher cumulative odds of reporting more frequent SSB and ASB consumption than infrequent viewers across all strata. The proportional-odds assumption was violated only for SSB consumption among high school students.

## 4. Discussion

The present study examined the associations between food-related online media viewing and SSB and ASB consumption among Korean adolescents and whether these associations differed by sex and school level. Overall, adolescents who frequently viewed food-related online media had higher odds of both SSB and ASB consumption than those who infrequently viewed such media. In particular, the associations for SSBs, were more pronounced among boys than girls, and were stronger among middle school students than high school students. For ASBs, the associations were stronger among boys, while the school-level pattern varied according to the frequency of beverage consumption.

In this study, we report that frequent viewers of food-related online media were associated with higher odds of both SSB and ASB consumption among adolescents, with generally stronger associations among boys, particularly at the highest levels of food-related online media viewing frequency. Although the mechanisms underlying these sex differences remain unclear, they may reflect differences in how boys and girls engage with food-related digital media and respond to messages surrounding food, health, and body image. Previous studies have reported that food-related online media viewing is associated not only with unhealthy dietary behaviors, including greater SSB consumption [12,25], but also with greater weight-control attempts and body image concerns among adolescents [6,12,25,47]. Our findings extend the previous literature by demonstrating that the associations between food-related online media viewing and beverage consumption are not limited to SSBs [11,12,17] but also include ASBs, suggesting that food-related online media viewing may be associated with a broader spectrum of beverage choices than previously recognized.

Notably, contrary to our a priori hypothesis that associations with ASB consumption would be stronger among girls, these associations were generally stronger among boys. This unexpected pattern may reflect growing concerns about muscularity, physique, and fitness, together with greater engagement in body composition-oriented dietary practices among boys [48,49,50]. However, the higher ASB consumption observed among frequent food-related online media viewers should not be interpreted as evidence of healthier beverage choices, as the long-term health effects of ASBs remain uncertain [26,31]. Together, these findings suggest that adolescents who frequently view food-related online media may simultaneously consume SSBs for hedonic reasons while choosing ASBs as perceived lower-sugar alternatives, reflecting competing dietary motivations [47].

Associations between food-related online media viewing frequency and beverage consumption also differed according to school level and beverage type. Frequent viewing was associated with higher odds of SSB consumption among both middle and high school students, although the associations were generally stronger among middle school students, particularly at higher levels of SSB consumption. These findings suggest that younger adolescents may be especially susceptible to external food cues and social modeling during a developmental period in which self-regulatory capacities are still maturing [9,10].

For ASB consumption, however, our a priori hypothesis that associations would be stronger among high school students was not consistently supported. Although the associations were somewhat stronger among high school students at occasional frequencies (3–4 and 5–6 times per week), they became stronger among middle school students at the highest consumption frequency (≥7 times per week). Thus, rather than reflecting a simple developmental gradient, this pattern suggests that the factors related to food-related online media viewing with ASB consumption may differ according to both developmental stage and the frequency of ASB consumption. Because ASB may be consumed for multiple reasons, including taste preferences, sugar reduction, and weight-control intentions, future longitudinal studies incorporating measures of body image, dieting behaviors, and beverage-related motivations are needed to determine whether food-related online media viewing primarily reinforces existing beverage preferences or actively shapes beverage choices throughout adolescence.

The sensitivity analyses further suggested that these associations were not limited to frequent viewers. When intermediate viewers were included in the analysis, intermediate viewers also showed higher odds of reporting more frequent SSB and ASB consumption across several consumption categories and strata. This pattern may suggest a graded association between food-related online media viewing and beverage consumption, although the cross-sectional design precludes conclusions regarding temporality or a dose–response relationship.

Although the mechanisms underlying the associations between food-related online media viewing and beverage consumption remain speculative, several biologically and behaviorally plausible pathways may explain these findings. *Mukbang* or *cookbang* videos frequently depict highly palatable foods and beverages, potentially eliciting cue-induced cravings and strengthening reward-related responses to food cues through repeated exposure and observational learning [5,8,12,15,17]. Within the framework of dual-process models of eating behavior [34], such exposure may activate impulsive, reward-driven processes that increase motivation to consume energy-dense foods and SSBs, while simultaneously activating more reflective processes related to body weight and calorie reduction. Previous studies have reported that frequent food-related online media viewing is associated with body image concerns and weight-control behaviors among adolescents [6,47], suggesting that some viewers may substitute ASBs for SSBs as perceived lower-sugar alternatives.

While SSB consumption may reflect cue-induced reward seeking and increased desire for highly palatable beverages, the concurrent association with ASB consumption suggests that some adolescents may simultaneously pursue calorie-conscious beverage choices. This observed association may also reflect broader behavioral patterns rather than the direct effect of food-related online media viewing alone. Consistent with previous KYRBS findings showing that SSB consumption clusters with multiple unhealthy lifestyle behaviors [51], food-related online media viewing may represent just one component of a broader constellation of digital media behaviors associated with unhealthy beverage consumption. Although the magnitude of these associations varied somewhat by sex and school level, the overall pattern reinforces this shared behavioral clustering among Korean adolescents.

However, these findings should be interpreted in light of several limitations. First, we used data from the Korea Youth Risk Behavior Survey, a large national surveillance survey of Korean adolescents. Because the primary analysis excluded intermediate viewers to maximize the contrast between the lowest and highest exposure groups, the primary analytic sample did not include the full distribution of food-related online media viewing among Korean adolescents. Intermediate viewers, accounting for approximately 44% of the complete-case analytic sample, were excluded from the primary analysis. Therefore, the primary findings may not fully represent associations across the broader distribution of food-related online media viewing among Korean adolescents. In addition, the exclusion of participants with missing data may have introduced selection bias if missingness was related to food-related online media viewing, beverage consumption, or other study characteristics. Furthermore, the cross-sectional design precludes causal inference, and reverse causation remains possible. The temporal ordering of some covariates in relation to the exposure and outcomes could not be clearly determined, and some may lie on the causal pathway or represent consequences of the exposure or outcome. Longitudinal studies are needed to clarify the temporal and causal relationships among food-related online media viewing, beverage consumption, and these covariates. Second, another concerning limitation is the operationalization of food-related online media exposure. The KYRBS assesses *mukbang* and *cookbang* viewing jointly; therefore, we were unable to distinguish between these genres in the present analysis. Although both formats involve prominent food-related visual and social cues, they may differ in content, viewer motivations, and their potential associations with dietary behaviors. Consequently, combining them into a single exposure measure may have obscured format-specific associations or otherwise altering the observed associations. Future studies using measures that separately assess *mukbang* and *cookbang* exposure are needed to determine whether these genres have distinct relationships with beverage consumption. Additionally, the survey did not collect information on the characteristics of food-related online media viewing, such as the types of foods and beverages portrayed, the platforms used, viewing duration, or the extent to which adolescents actively engaged with the content or creators. These factors may modify the association between this exposure and beverage consumption and should be examined in future research. Moreover, food-related online media viewing was assessed over the previous 12 months, whereas beverage consumption reflected intake during the past 7 days. Consequently, because food-related online media viewing and beverage consumption were assessed over different time frames, it is difficult to determine whether food-related online media viewing influences beverage consumption over shorter or longer periods. Third, beverage intake was assessed using a single-item frequency measure from the KYRBS rather than a validated multi-item dietary assessment instrument. Although this approach is consistent with the population-level surveillance purpose of the KYRBS and has been used in previous studies of Korean adolescents [25,32], it does not provide information on the SSB or ASB volume or total intake and may be subject to measurement error. Therefore, the findings should be interpreted as reflecting the reported frequency of beverage consumption rather than precise estimates of beverage intake. Relatedly, the operational definitions and examples used to classify SSBs and ASBs in the 2025 KYRBS differ from those used in other studies, which may limit the direct comparability of our findings. In addition, ASB consumption was based on the survey-defined “zero beverage” category, and the survey did not collect information on the specific types or combinations of sweeteners contained in individual beverages. Therefore, we could not independently verify the sweetener composition of each beverage or distinguish among specific sweeteners; hence the ASB findings should be interpreted within the operational definition used by the 2025 KYRBS. Exposure, outcome, and covariates were self-reported and may be subject to misclassification. Residual confounding from unmeasured factors, including parental beverage consumption, the home food environment, peer influences, and other food-related digital media viewing, cannot be excluded.

Nevertheless, this study also has several strengths. First, it assessed both SSBs and ASBs, whereas nationally representative studies examining ASB consumption among adolescents remain limited. The large nationally representative sample provided sufficient statistical power for stratified analyses while allowing for one of the first population-based examinations of food-related online media viewing and ASB consumption among adolescents.

The findings of this study have several implications for research, clinical practice, and public health. As adolescents increasingly obtain food-related information and entertainment through digital media, food-related online media viewing may represent a novel and potentially modifiable behavioral correlate of unhealthy beverage consumption. Furthermore, because significant effect modification by sex and school level was observed, future studies should examine the behavioral, psychosocial, and environmental factors that may explain these subgroup differences. Such work may help identify adolescents who are particularly susceptible to the associations between food-related digital media viewing and beverage consumption. Healthcare providers, educators, and parents should be aware that frequent engagement with food-related online content is associated with beverage consumption patterns among adolescents. However, the findings do not establish that reducing exposure to food-related online media would reduce beverage consumption. Longitudinal and intervention studies are needed to determine whether changes in exposure or engagement with food-related online media are associated with subsequent changes in beverage consumption. Future research should also consider the variations by sex and school level when developing and evaluating targeted prevention strategies.

## 5. Conclusions

Frequent food-related online media viewing was associated with more frequent consumption of both SSB and ASB among Korean adolescents. The magnitude of these associations differed by sex and school level, suggesting that demographic characteristics may modify the relationship between food-related digital media viewing and beverage consumption. Longitudinal studies are needed to clarify the direction of these associations and elucidate the mechanisms through which food-related online media viewing may influence adolescents’ dietary behaviors.

## Figures and Tables

**Table 1:** Verbatim Questions, Response Options, and Cutoff Points of Independent, Dependent, and Moderator Variables.

Variables	Questions	Response Options	Cutoff Points
Independent variable			
*Mukbang*	Over the past 12 months, how often have you watched *mukbang* (eating broadcast) or *cookbang* (cooking broadcast)?	Not at all Less than once per month Once to three times per month Once or twice per week Three to four times per week Five to six times per week Daily (7 times or more per week)	Frequent *mukbang* viewing: ≥5 times/week Infrequent *mukbang* viewing: ≤once per month
Dependent variables			
Sugar-sweetened beverage (SSB) consumption	Over the past 7 days, how often have you drunk sugar-sweetened beverages? Examples: carbonated beverages (excluding zero beverages), energy drinks (e.g., Hot6, Red Bull, Bacchus), sports drinks (e.g., Gatorade, Pocari Sweat), fruit beverages (e.g., Sunkist Drink, Capri-Sun), coffee beverages (e.g., coffee mix, Let’s Be), and sweetened milk (e.g., chocolate and banana milk)	Did not drink during the past 7 days 1 to 2 times per week 3 to 4 times per week 5 to 6 times per week Once per day Twice per day Three or more times per day	Did not drink over the past 7 days 1 to 2 times per week 3 to 4 times per week 5 to 6 times per week Once or more per day
Artificially sweetened beverage (ASB) consumption	Over the past 7 days, how often have you drunk zero-calorie (sugar-free) beverages? Zero beverages refer to beverages that use artificial sweeteners instead of sugar to provide sweetness but contain almost no calories,” followed by examples such as Coke Zero, Sprite Zero, Powerade Zero, and Toreta Zero	Did not drink during the past 7 days 1 to 2 times per week 3 to 4 times per week 5 to 6 times per week Once per day Twice per day Three or more times per day	Did not drink over the past 7 days 1 to 2 times per week 3 to 4 times per week 5 to 6 times per week Once or more per day
Moderators			
Sex	What was your sex assigned at birth?	Male Female	
School level	What grade are you in?	Middle school 1st grade Middle school 2nd grade Middle school 3rd grade High school 1st grade High school 2nd grade High school 3rd grade	Middle school: middle school 1st grade-3rd grade High school: high school 1st grade-3rd grade
Covariates			
Age (years)	How old are you?		
Academic performance	During the past 12 months, how would you describe your academic performance?	High Medium to high Medium Low to medium Low	
Perceived socioeconomic status	What is the financial situation of your household?	High Medium to high Medium Low to medium Low	
Perceived health	How would you describe your health status?	Extremely healthy Slightly healthy Neither healthy nor unhealthy Slightly unhealthy Extremely unhealthy	
BMI percentile	What is your most recently measured height? What is your most recently measured weight?	Body weight status was classified according to age- and sex-specific BMI percentiles based on the 2017 Korean National Growth Charts for Children and Adolescents	
Weight-control effort	Over the past 30 days, have you made any effort to control your body weight?	Did not place any effort Attempted to lose weight Attempted to gain weight Attempted to maintain weight	
Physical activity	Over the past 7 days, on how many days did you engage in physical activity of any kind that increased your heart rate or made you breathe hard for a total of at least 60 min per day?	None 1 day/week 2 days/week 3 days/week 4 days/week 5 days/week 6 days/week 7 days/week	
Sleep	Over the past 7 days, what time have you usually gone to bed and woken up?	Weekdays: bedtime and wake-up time Weekends: bedtime and wake-up time	
Alcohol use	Over the past 30 days, on how many days did you drink at least one alcoholic drink?	Never drank alcohol (lifetime) None 1–2 days/month 3–5 days/month 6–9 days/month 10–19 days/month 20–29 days/month Daily	
Smoking status	Over the past 30 days, on how many days did you smoke?	Never smoked (lifetime) None 1–2 days/month 3–5 days/month 6–9 days/month 10–19 days/month 20–29 days/month Daily	
Screen time of smartphone	Over the past 7 days, on average, how many hours per day did you use a smartphone?	Weekdays: did not use/used; average daily smartphone use time Weekends: did not use/used; average daily smartphone use time	

**Table 2:** General Characteristics of Study Participants by Frequency of Food-Related Online Media Viewing.

Variables	Infrequent Food-Related Online Media Viewers (*n* = 22,750)	Frequent Food-Related Online Media Viewers (*n* = 4354)	Total (*n* = 27,104)	*p* Value
Sex ^1^				
Male	14,614 (64.6)	1342 (30.1)	15,956 (59.1)	<0.001
Female	8136 (35.4)	3012 (69.9)	11,148 (40.9)	
Age (yr) ^2^	14.95 ± 1.77	15.14 ± 1.78	14.98 ± 1.78	<0.001
School level ^1^				
Middle school	11,856 (51.4)	2108 (47.3)	13,964 (50.7)	<0.001
High school	10,894 (48.6)	2246 (52.7)	13,140 (49.3)	
Academic performance ^1^				
High	3353 (15.1)	393 (8.9)	3746 (14.1)	<0.001
Medium to high	6182 (27.6)	926 (21.1)	7108 (26.6)	
Medium	6313 (27.6)	1281 (29.8)	7594 (27.9)	
Low to Medium	4887 (21.1)	1212 (27.6)	6099 (22.1)	
Low	2015 (8.7)	542 (12.7)	2557 (9.3)	
Perceived socioeconomic status ^1^				
High	2743 (12.6)	447 (10.8)	3190 (12.3)	<0.001
Medium to high	7454 (33.5)	1237 (28.5)	8691 (32.7)	
Medium	10,214 (44.1)	2128 (48.5)	12,342 (44.8)	
Low to Medium	1958 (8.2)	445 (10.2)	2403 (8.5)	
Low	381 (1.5)	97 (2.1)	478 (1.6)	
Perceived health ^1^				
Extremely healthy	5885 (25.7)	900 (20.3)	6785 (24.9)	<0.001
Healthy	10,063 (44.2)	1923 (44.2)	11,986 (44.2)	
Average	4976 (22.0)	1099 (25.6)	6075 (22.5)	
Unhealthy	1727 (7.6)	412 (9.5)	2139 (7.9)	
Extremely unhealthy	99 (0.4)	20 (0.4)	119 (0.4)	
Weight-control effort (over the past 30 days) ^1^				
Did not place any effort	11,638 (51.7)	1527 (35.4)	13,165 (49.1)	<0.001
Attempted to lose weight	6649 (28.6)	2030 (46.4)	8679 (31.4)	
Attempted to gain weight	2024 (8.9)	246 (5.5)	2270 (8.3)	
Attempted to maintain weight	2439 (10.8)	551 (12.7)	2990 (11.1)	
Physical activity (times/past 7 days) ^1^				
None	7251 (32.1)	1552 (36.5)	8803 (32.8)	<0.001
1 day/week	3037 (13.4)	592 (13.1)	3629 (13.3)	
2 days/week	3400 (14.9)	626 (14.2)	4026 (14.8)	
3 days/week	3191 (14.2)	543 (12.6)	3734 (13.9)	
4 days/week	1763 (7.7)	300 (7.0)	2063 (7.6)	
5 days/week	1671 (7.3)	276 (6.3)	1947 (7.1)	
6 days/week	628 (2.8)	95 (2.1)	723 (2.7)	
7 days/week	1809 (7.7)	370 (8.1)	2179 (7.8)	
Smoking status (past 30 days) ^1^				
Never smoked (lifetime)	21,344 (94.0)	4000 (91.5)	25,344 (93.6)	<0.001
None	756 (3.3)	187 (4.5)	943 (3.5)	
1–2 days/month	109 (0.5)	29 (0.7)	138 (0.5)	
3–5 days/month	52 (0.2)	11 (0.3)	63 (0.2)	
6–9 days/month	31 (0.1)	10 (0.2)	41 (0.1)	
10–19 days/month	78 (0.4)	17 (0.4)	95 (0.4)	
20–29 days/month	62 (0.2)	20 (0.5)	82 (0.3)	
Daily	318 (1.4)	80 (1.9)	398 (1.5)	
Lifetime alcohol use (past 30 days) ^1^				
Never drank alcohol (lifetime)	17,024 (74.7)	2979 (68.0)	20,003 (73.6)	<0.001
None	4098 (18.1)	956 (22.2)	5054 (18.7)	
1–2 days/month	990 (4.4)	255 (5.8)	1245 (4.6)	
3–5 days/month	317 (1.4)	84 (2.0)	401 (1.5)	
6–9 days/month	163 (0.7)	44 (1.2)	207 (0.8)	
10–19 days/month	96 (0.4)	21 (0.4)	117 (0.4)	
20–29 days/month	46 (0.2)	10 (0.2)	56 (0.2)	
Daily	16 (0.1)	5 (0.1)	21 (0.1)	
BMI percentile	50.90 ± 28.3	49.04 ± 28.7	50.60 ± 38.2	0.001
Sleep (hrs) ^2^	6.99 ± 1.58	6.72 ± 1.65	6.94 ± 1.61	<0.001
Screen time (hrs) ^2^	4.51 ± 2.91	5.85 ± 3.22	4.72 ± 3.04	<0.001

^1^ Values are presented as *n* (weighted %). ^2^ Values are presented as mean ± standard deviation.

**Table 3:** Odds Ratios (95% CIs) for Sugar-Sweetened Beverage (SSB) and Artificially Sweetened Beverage (ASB) Consumption Frequencies According to Food-Related Online Media Viewing Frequency, Stratified by Sex.

			SSB ^1^				ASB ^2^			
			OR (95% CI) ^3^				OR (95% CI) ^3^			
			1–2 Times	3–4 Times	5–6 Times	≥7 Times	1–2 Times	3–4 Times	5–6 Times	≥7 Times
Boys	Model 1 ^4^	Infrequent viewers (*n* = 14,614)	1.00 (ref)	1.00 (ref)	1.00 (ref)	1.00 (ref)	1.00 (ref)	1.00 (ref)	1.00 (ref)	1.00 (ref)
		Frequent viewers (*n* = 1342)	0.74 (0.58–0.95)	0.95 (0.75–1.21)	1.28 (0.99–1.64)	1.79 (1.40–2.28)	1.46 (1.26–1.69)	2.23 (1.85–2.69)	2.71 (2.07–3.54)	3.86 (3.08–4.82)
	Model 2 ^5^	Infrequent viewers (*n* = 14,614)	1.00 (ref)	1.00 (ref)	1.00 (ref)	1.00 (ref)	1.00 (ref)	1.00 (ref)	1.00 (ref)	1.00 (ref)
		Frequent viewers (*n* = 1342)	0.78 (0.61–1.00)	1.00 (0.78–1.28)	1.38 (1.06–1.79)	1.79 (1.39–2.31)	1.36 (1.16–1.59)	2.02 (1.66–2.46)	2.37 (1.83–3.06)	3.98 (3.23–4.91)
Girls	Model 1 ^4^	Infrequent viewers (*n* = 8136)	1.00 (ref)	1.00 (ref)	1.00 (ref)	1.00 (ref)	1.00 (ref)	1.00 (ref)	1.00 (ref)	1.00 (ref)
		Frequent viewers (*n* = 3012)	0.96 (0.83–1.11)	1.21 (1.03–1.41)	1.60 (1.32–1.92)	1.57 (1.31–1.88)	1.34 (1.22–1.47)	1.81 (1.55–2.11)	1.84 (1.41–2.41)	2.44 (1.88–3.16)
	Model 2 ^5^	Infrequent viewers (*n* = 8136)	1.00 (ref)	1.00 (ref)	1.00 (ref)	1.00 (ref)	1.00 (ref)	1.00 (ref)	1.00 (ref)	1.00 (ref)
		Frequent viewers (*n* = 3012)	0.93 (0.79–1.08)	1.11 (0.95–1.31)	1.44 (1.19–1.74)	1.37 (1.14–1.65)	1.24 (1.12–1.37)	1.52 (1.31–1.77)	1.62 (1.26–2.08)	2.20 (1.78–2.71)

Reference category for SSB and ASB consumption was “never”. ^1^ SSB: sugar-sweetened beverages (times over the past 7 days). ^2^ ASB: zero-calorie (sugar-free) beverages (times over the past 7 days). ^3^ Values are presented as odds ratios (OR) and 95% confidence intervals (CIs). ^4^ Model 1: crude model. ^5^ Model 2: adjusted for variables that include age, academic performance, perceived socioeconomic status, perceived health, weight-control effort, physical activity, sleep duration, smoking status, alcohol use, BMI percentile, and smartphone screen time.

**Table 4:** Odds Ratios (95% CIs) for Sugar-Sweetened Beverage (SSB) and Artificially Sweetened Beverage (ASB) Consumption Frequencies According to Food-Related Online Media Viewing Frequency, Stratified by School Level.

			SSB ^1^				ASB ^2^			
			OR (95% CI) ^3^				OR (95% CI) ^3^			
			1–2 Times	3–4 Times	5–6 Times	≥7 Times	1–2 Times	3–4 Times	5–6 Times	≥7 Times
Middle School	Model 1 ^4^	Infrequent viewers (*n* = 11,856)	1.00 (ref)	1.00 (ref)	1.00 (ref)	1.00 (ref)	1.00 (ref)	1.00 (ref)	1.00 (ref)	1.00 (ref)
		Frequent viewers (*n* = 2108)	0.98 (0.81–1.19)	1.20 (0.98–1.47)	1.50 (1.20–1.88)	1.85 (1.52–2.26)	1.22 (1.09–1.36)	1.39 (1.16–1.65)	1.62 (1.21–2.17)	2.53 (2.00–3.20)
	Model 2 ^5^	Infrequent viewers (*n* = 11,856)	1.00 (ref)	1.00 (ref)	1.00 (ref)	1.00 (ref)	1.00 (ref)	1.00 (ref)	1.00 (ref)	1.00 (ref)
		Frequent viewers (*n* = 2108)	0.94 (0.77–1.16)	1.25 (1.00–1.55)	1.80 (1.41–2.30)	2.07 (1.66–2.57)	1.26 (1.12–1.42)	1.43 (1.19–1.70)	1.66 (1.23–2.21)	3.45 (2.77–4.31)
High School	Model 1 ^4^	Infrequent viewers (*n* = 10,894)	1.00 (ref)	1.00 (ref)	1.00 (ref)	1.00 (ref)	1.00 (ref)	1.00 (ref)	1.00 (ref)	1.00 (ref)
		Frequent viewers (*n* = 2246)	0.82 (0.70–0.96)	0.85 (0.73–1.00)	0.98 (0.81–1.18)	1.02 (0.85–1.23)	1.29 (1.15–1.45)	1.69 (1.47–1.96)	1.73 (1.39–2.17)	1.90 (1.53–2.36)
	Model 2 ^5^	Infrequent viewers (*n* = 10,894)	1.00 (ref)	1.00 (ref)	1.00 (ref)	1.00 (ref)	1.00 (ref)	1.00 (ref)	1.00 (ref)	1.00 (ref)
		Frequent viewers (*n* = 2246)	0.86 (0.72–1.02)	0.97 (0.82–1.15)	1.21 (0.99–1.49)	1.28 (1.05–1.56)	1.28 (1.13–1.44)	1.92 (1.64–2.26)	1.93 (1.52–2.46)	2.28 (1.81–2.88)

Reference category for SSB and ASB consumption was “never”. ^1^ SSB: sugar-sweetened beverages (times over the past 7 days). ^2^ ASB: zero-calorie (sugar-free) beverages (times over the past 7 days). ^3^ Values are presented as odds ratios (OR) and 95% confidence intervals (CIs). ^4^ Model 1: crude model. ^5^ Model 2: adjusted for variables that include age, sex, academic performance, perceived socioeconomic status, perceived health, weight-control effort, physical activity, sleep duration, smoking status, alcohol use, BMI percentile, and smartphone screen time.

## Data Availability

Data described in the manuscript are publicly and freely available without restriction at the Korea Centers for Disease Control and Prevention repository (https://www.kdca.go.kr/yhs/ (Accessed on 8 July 2026)).

## Supplementary Materials

The following supporting information can be downloaded at: https://www.mdpi.com/article/10.3390/nu18203402/s1, Table S1: Odds Ratios (95% CIs) for Sugar-Sweetened Beverage (SSB) and Artificially Sweetened Beverage (ASB) Consumption Frequencies According to Food-Related Online Media Viewing Frequency, Stratified by Sex; Table S2: Odds Ratios (95% CIs) for Sugar-Sweetened Beverage (SSB) and Artificially Sweetened Beverage (ASB) Consumption Frequencies According to Food-Related Online Media Viewing Frequency, Stratified by School Level; Table S3: Ordinal Logistic Regression (Proportional Odds Model) for the Association Between Food-Related Online Media Viewing Frequency.
