# Supplementary material for: Food-Related Online Media Viewing (Mukbang and Cookbang) and Sugar-Sweetened Beverage and Artificially Sweetened Beverage Consumption Among Korean Adolescents: Using the 2025 Korea Youth Risk Behavior Survey
Source: Nutrients. 2026 Oct 16;18(20):3402. doi: 10.3390/nu18203402 (PMC13708191; doi:10.3390/nu18203402)
Supplement: Supplementary file 1 [file nutrients-18-03402-s001.zip › nutrients-4609764-supplementary.pdf]

Supplementary materials

Table S1. Odds Ratios (95% CIs) for Sugar-Sweetened Beverage (SSB) and Artificially Sweetened Beverage (ASB) Consumption Frequencies According to Food-Related Online Media Viewing Frequency, Stratified by Sex

			SSB[1]				ASB[2]			
			OR (95% CI)[3]				OR (95% CI)[3]			
			1-2 times	3-4 times	5-6 times	≥7 times	1-2 times	3-4 times	5-6 times	≥7 times
Boys	Model 1[4]	Infrequent viewers (n=14,614)	1.00 (ref)	1.00 (ref)	1.00 (ref)	1.00 (ref)	1.00 (ref)	1.00 (ref)	1.00 (ref)	1.00 (ref)
		Intermediate viewers (n=8,906)	1.53 (1.34-1.75)	1.95 (1.71-2.23)	1.97 (1.71-2.28)	1.82 (1.56-2.12)	1.37 (1.29-1.46)	1.61 (1.47-1.76)	1.76 (1.53-2.03)	1.48 (1.30-1.68)
		Frequent viewers (n=1,342)	0.74 (0.58-0.95)	0.95 (0.75-1.21)	1.28 (0.99-1.64)	1.79 (1.40-2.28)	1.46 (1.26-1.69)	2.23 (1.85-2.69)	2.71 (2.07-3.54)	3.86 (3.08-4.82)
	Model 2[5]	Infrequent viewers (n=14,614)	1.00 (ref)	1.00 (ref)	1.00 (ref)	1.00 (ref)	1.00 (ref)	1.00 (ref)	1.00 (ref)	1.00 (ref)
		Intermediate viewers (n=8,906)	1.53 (1.34-1.75)	1.95 (1.70-2.23)	1.99 (1.72-2.31)	1.85 (1.58-2.16)	1.32 (1.23-1.41)	1.52 (1.39-1.66)	1.64 (1.41-1.89)	1.42 (1.25-1.63)
		Frequent viewers (n=1,342)	0.78 (0.61-1.00)	1.00 (0.78-1.28)	1.35 (1.04-1.76)	1.79 (1.39-2.29)	1.39 (1.20-1.62)	2.00 (1.66-2.42)	2.25 (1.72-2.96)	3.18 (2.52-4.02)
Girls	Model 1[4]	Infrequent viewers (n=8,136)	1.00 (ref)	1.00 (ref)	1.00 (ref)	1.00 (ref)	1.00 (ref)	1.00 (ref)	1.00 (ref)	1.00 (ref)
		Intermediate viewers (n=12,476)	1.32 (1.19-1.46)	1.57 (1.40-1.75)	1.80 (1.58-2.06)	1.37 (1.20-1.55)	1.36 (1.28-1.45)	1.37 (1.23-1.52)	1.42 (1.18-1.72)	1.21 (0.97-1.52)
		Frequent viewers (n=3,012)	0.96 (0.83-1.11)	1.21 (1.03-1.41)	1.60 (1.32-1.92)	1.57 (1.31-1.88)	1.34 (1.22-1.47)	1.81 (1.55-2.11)	1.84 (1.41-2.41)	2.44 (1.88-3.16)
	Model 2[5]	Infrequent viewers (n=8,136)	1.00 (ref)	1.00 (ref)	1.00 (ref)	1.00 (ref)	1.00 (ref)	1.00 (ref)	1.00 (ref)	1.00 (ref)
		Intermediate viewers (n=12,476)	1.29 (1.17-1.43)	1.51 (1.34-1.69)	1.73 (1.51-1.98)	1.34 (1.18-1.53)	1.31 (1.23-1.39)	1.26 (1.12-1.41)	1.37 (1.13-1.65)	1.18 (0.95-1.48)

	Frequent viewers (n=3,012)	0.98 (0.84-1.14)	1.16 (0.98-1.36)	1.47 (1.21-1.77)	1.40 (1.16-1.69)	1.26 (1.15-1.39)	1.50 (1.28-1.76)	1.55 (1.18-2.04)	2.03 (1.55-2.65)

Reference category for SSB and ASB consumption was "never"

[1]SSB: sugar-sweetened beverages (times over the past 7 days).

[2]ASB: zero-calorie (sugar-free) beverages (times over the past 7 days).

[3]Values are presented as odds ratios (OR) and 95% confidence intervals (CIs).

[4]Model 1: crude model.

[5]Model 2: adjusted for variables that include age, sex, academic performance, perceived socioeconomic status, perceived health, weight-control effort, physical activity, sleep duration, smoking status, alcohol use, BMI percentile, and smartphone screen time

Table S2. Odds Ratios (95% CIs) for Sugar-Sweetened Beverage (SSB) and Artificially Sweetened Beverage (ASB) Consumption Frequencies According to Food-Related Online Media Viewing Frequency, Stratified by School Level

			SSB[1]				ASB[2]			
			OR (95% CI)[3]				OR (95% CI)[3]			
			1-2 times	3-4 times	5-6 times	≥7 times	1-2 times	3-4 times	5-6 times	≥7 times
Middle School	Model 1[4]	Infrequent viewers (n=11,856)	1.00 (ref)	1.00 (ref)	1.00 (ref)	1.00 (ref)	1.00 (ref)	1.00 (ref)	1.00 (ref)	1.00 (ref)
		Intermediate viewers (n=11,429)	1.45 (1.29-1.62)	1.78 (1.57-2.00)	1.76 (1.55-2.00)	1.49 (1.30-1.71)	1.25 (1.18-1.32)	1.19 (1.09-1.31)	1.39 (1.18-1.62)	1.16 (0.99-1.36)
		Frequent viewers (n=2,108)	0.98 (0.81-1.19)	1.20 (0.98-1.47)	1.50 (1.20-1.88)	1.85 (1.52-2.26)	1.22 (1.09-1.36)	1.39 (1.16-1.65)	1.62 (1.21-2.17)	2.53 (2.00-3.20)
	Model 2[5]	Infrequent viewers (n=11,856)	1.00 (ref)	1.00 (ref)	1.00 (ref)	1.00 (ref)	1.00 (ref)	1.00 (ref)	1.00 (ref)	1.00 (ref)
		Intermediate viewers (n=11,429)	1.41 (1.26-1.58)	1.87 (1.65-2.11)	1.96 (1.73-2.23)	1.73 (1.51-1.99)	1.28 (1.20-1.36)	1.27 (1.14-1.40)	1.51 (1.28-1.79)	1.41 (1.18-1.67)
		Frequent viewers (n=2,108)	0.97 (0.80-1.19)	1.30 (1.05-1.60)	1.72 (1.36-2.18)	2.09 (1.68-2.59)	1.27 (1.13-1.42)	1.49 (1.24-1.78)	1.68 (1.23-2.29)	2.98 (2.30-3.87)
High School	Model 1[4]	Infrequent viewers (n=10,894)	1.00 (ref)	1.00 (ref)	1.00(ref)	1.00 (ref)	1.00 (ref)	1.00 (ref)	1.00 (ref)	1.00 (ref)
		Intermediate viewers (n=9,953)	1.30 (1.16-1.45)	1.36 (1.22-1.52)	1.43 (1.25-1.63)	1.18 (1.05-1.34)	1.33 (1.25-1.42)	1.40 (1.28-1.53)	1.40 (1.22-1.61)	1.08 (0.94-1.24)
		Frequent viewers (n=2,246)	0.82 (0.70-0.96)	0.85 (0.73-1.00)	0.98 (0.81-1.18)	1.02 (0.85-1.23)	1.29 (1.15-1.45)	1.69 (1.47-1.96)	1.73 (1.39-2.17)	1.90 (1.53-2.36)
	Model 2[5]	Infrequent viewers (n=10,894)	1.00 (ref)	1.00 (ref)	1.00 (ref)	1.00 (ref)	1.00 (ref)	1.00 (ref)	1.00 (ref)	1.00 (ref)
		Intermediate viewers (n=9,953)	1.36 (1.21-1.52)	1.51 (1.35-1.69)	1.67 (1.46-1.91)	1.45 (1.27-1.65)	1.35 (1.26-1.44)	1.57 (1.42-1.72)	1.58 (1.37-1.83)	1.31 (1.13-1.51)

	Frequent viewers (n=2,246)	0.91 (0.77-1.08)	1.03 (0.87-1.21)	1.27 (1.04-1.54)	1.34 (1.11-1.62)	1.33 (1.18-1.49)	1.98 (1.69-2.31)	2.03 (1.60-2.56)	2.32 (1.83-2.93)

Reference category for SSB and ASB consumption was “never”

[1]SSB: sugar-sweetened beverages (times over the past 7 days).

[2]ASB: zero-calorie (sugar-free) beverages (times over the past 7 days).

[3]Values are presented as odds ratios (OR) and 95% confidence intervals (CIs).

[4]Model 1: crude model.

[5]Model 2: adjusted for variables that include age, sex, academic performance, perceived socioeconomic status, perceived health, weight-control effort, physical activity, sleep duration, smoking status, alcohol use, BMI percentile, and smartphone screen time

Table S3. Ordinal Logistic Regression (proportional odds model) For the Association Between Food-Related Online Media Viewing Frequency

		Cumulative OR (95% CI)[1]	Test of parallel lines[1]
Boys	SSB[3]	1.63(1.45-1.83)	F=0.687, p=0.903
	ASB[4]	1.90(1.68-2.14)	F=0.026, p=0.999
Girls	SSB	1.29(1.20-1.39)	F=0.176, p>0.999
	ASB	1.40(1.28-1.52)	F<0.001, p>0.999
Middle school	SSB	1.60(1.46-1.76)	F=0.209, p>0.999
	ASB	1.48(1.34-1.64)	F=0.038, p>0.999
High school	SSB	1.25(1.15-1.37)	F=3.199, p<0.001
	ASB	1.63(1.47-1.80)	F=0.033, p>0.999

[1]Cumulative odds of a higher consumption-frequency category for frequent viewers compared with infrequent viewers (reference). Values are presented as odds ratios (OR) and 95% confidence intervals (CIs). Adjusted for age, academic performance, perceived socioeconomic status, perceived health status, weight control effort, physical activity, alcohol use, smoking status, BMI percentile, sleep duration, and smartphone screen time; sex was additionally adjusted for in school-level strata.

[2]Wald F test for the proportional odds assumption; $p < 0.05$ indicates violation of the assumption.

[3]SSB: sugar-sweetened beverages (times over the past 7 days).

[4]ASB: zero-calorie (sugar-free) beverages (times over the past 7 days).
